# Intracardiac or transesophageal echocardiography for left atrial appendage occlusion: an updated systematic review and meta-analysis

**DOI:** 10.1007/s10554-025-03330-z

**Published:** 2025-01-22

**Authors:** Eirini Beneki, Kyriakos Dimitriadis, Panagiotis Theofilis, Nikolaos Pyrpyris, Panayiotis Iliakis, Argyro Kalompatsou, Panagiotis Kostakis, Markos Koukos, Stergios Soulaidopoulos, Georgios Tzimas, Konstantinos Tsioufis, Patrizio Lancellotti, Constantina Aggeli

**Affiliations:** 1 First Cardiology Department, School of Medicine, Hippokration General Hospital, National and Kapodistrian University of Athens, Athens, Greece; 2 Department of Cardiology, Lausanne University Hospital and University of Lausanne, Lausanne, Switzerland; 3 Department of Cardiology, University Hospital Center, Liege, Belgium

**Keywords:** Intracardiac, Transesophageal, Echocardiography, Left atrial appendage, Occlusion

## Abstract

**Background:**

Intracardiac echocardiography (ICE) appears to be a potential alternative for percutaneous left atrial appendage occlusion (LAAO) to transesophageal echocardiography (TEE). Thus, a meta-analysis was performed comparing ICE vs. TEE for LAAO guidance.

**Methods:**

A comprehensive literature search was performed using MEDLINE, Scopus and Web of Science electronic databases from their inception to November 2023.

**Results:**

18 studies (124,230 patients) were included. Technical success was higher in ICE- compared to TEE-guidance (OR: 1.36, 95% CI 1.14 to 1.63, *p* = 0.006) and fewer devices employed (SMD: -0.22, 95% CI -0.43 to -0.01, *p* = 0.04, I2 = 62%). ICE guidance related with more pericardial effusion/tamponade and iatrogenic residual shunts (logRR: 0.62, 95% CI 0.36 to 0.89, *p* < 0.001 and RR: 1.53, 95% CI 1.12 to 2.09, *p* = 0.02, I2 = 1%, respectively). More vascular complications were noted in ICE group (logRR: 0.45, 95% CI 0.11 to 0.78, *p* = 0.009).

**Conclusion:**

ICE-guided imaging is an effective alternative to TEE in LAAO, as it shows better efficacy than TEE, considering technical success. However, the higher rates of adverse events should be carefully considered.

**Supplementary Information:**

The online version contains supplementary material available at 10.1007/s10554-025-03330-z.

## Introduction

Atrial fibrillation (AF) is the most frequent cardiac arrhythmia worldwide with a prevalence that is expected to increase in proportion among the elderly population over the following decades [1], and accounts for approximately 20–30% of ischemic strokes [2]. In non-valvular AF, nearly 90% of cardioembolic strokes originate from thrombus formation in the left atrial appendage (LAA) [3–5]. Long-term oral anticoagulation (OAC) is currently the standard treatment for stroke prevention in patients with AF, particularly when there is a high risk of ischemic stroke assessed most frequently using the CHA_2_DS_2_-VASc score [2, 6].

Two randomized controlled studies, PROTECT AF and PREVAIL [7–10], have proven the efficacy of LAA occlusion (LAAO) in preventing AF-related stroke and reducing major bleeding events compared with warfarin. Recent clinical studies demonstrated the non-inferiority of LAAO versus novel OACs in reducing thromboembolic and major bleeding events during long‐term follow‐up [11, 12]. The 2019 AHA/ACC/HRS guidelines [13] and 2020 ESC guidelines [14] for the diagnosis and management of AF both recommend LAAO to prevent stroke in patients with nonvalvular AF and contraindications to anticoagulation (IIb). LAAO has emerged as a safe and effective alternative preventive treatment to LAA thrombus formation and reduces the risk of thromboembolism among patients with absolute or relative contraindications to anticoagulation treatment [15, 16]. The commonly used devices for LAAO procedures include Watchman and Amplatzer Cardiac Plug (ACP) [17–20].

Pre-procedural planning needed for successful deployment of LAAO devices includes the utilization of cardiac computed tomography angiography (CCTA) and transesophageal echocardiography (TEE) to detect LAA thrombus and identify appendage morphology with the appropriate device sizing. TEE is most commonly utilized in conjunction with fluoroscopy for procedural guidance [21]. TEE has significant drawbacks including the frequent need for general anesthesia or deep sedation and longer procedural times [21]. In recently years, intracardiac echocardiography (ICE) has been proposed as an alternative to TEE for LAAO procedure guidance [22]. Studies comparing TEE with ICE for LAAO are few and most of them are single-center studies with a small number of patients, resulting in limited information about efficacy and safety outcomes. Therefore, the objective of this updated meta-analysis is to evaluate the available data comparing the outcome profile of TEE vs. ICE guidance for percutaneous LAAO.

## Methods

### Data source

We searched MEDLINE, Scopus, and Web of Science electronic databases from their inception to November 2023 for studies comparing ICE vs. TEE in LAAO. The present meta-analysis was performed in accordance with the Cochrane Collaboration and Preferred Reporting Items for Systematic Reviews and Meta‐Analyses (PRISMA) statements and the registered protocol is displayed in the PROSPERO database (CRD42024526878) [23].

### Search strategy

The relevant combinations of the medical subject heading (MeSH) terms used in searches included “atrial fibrillation” AND “left atrial appendage” AND “(closure OR occlusion)” AND “intracardiac echocardiography” AND “transesophageal echocardiography”. Studies were included regardless of their language and year of publication.

### Study selection

Studies were considered eligible if they met the following criteria: [1] human subjects aged ≥ 18 years undergoing LAAO under ICE or TEE guidance; and [2] studies compared the use of TEE vs. ICE to guide deployment of LAAO device [3], procedural characteristics and clinical outcomes were compared between the ICE-guided group and the TEE-guided group. Studies involving single-arm studies, case reports, editorials, reviews, and expert opinions, were excluded from the present analysis.

### Data extraction

Two investigators (E. B. and N. P.) independently screened all titles and abstracts, and manually searched the full text versions of all relevant studies that fulfilled the inclusion criteria. The reference lists of the retrieved articles were also independently reviewed. Any discrepancies between the two investigators were resolved by a third investigator (PI). Data on study characteristics, baseline characteristics of included patients, procedural characteristics and complications, and follow-up imaging were extracted for the present analysis.

### Quality assessment of studies

The Newcastle-Ottawa Scale [24] (Table 1) was used to appraise the quality of included studies.

**Table 1:** Qualitative evaluation of included studies using Newcastle- Ottawa Scale

Study	Selection (max 4 stars)	Comparability (max 2 stars)	Outcome (max 3 stars)
Frangieh et al.	****	*	**
Iwasawa et al.	****	*	*
Korsholm et al.	****	*	***
Reis et al.	****	*	***
Berti et al.	****	**	***
Kim et al.	****	**	***
Streb et al.	****	*	***
Hemam et al.	****	*	***
Nielsen- Kudsk et al.	****	**	***
Alkhouli et al.	****	**	***
Grazina et al.	***	*	***
Gianni et al.	****	*	***
Pommier et al.	****	*	***
Zahid et al.	****	**	**
Morcos et al.	****	**	**
Ferro et al.	****	*	***
Chu et al.	***	*	***
Pastormerlo et al.	***	*	**

### Primary and secondary outcomes

The primary efficacy outcome was technical success, defined as successful deployment and implantation of the device, using the selected imaging modality. The primary safety outcome was the occurrence of any reported procedure-related and device-related complication. Complications included: pericardial effusion (with and without tamponade), stroke/transient ischemic attack, device embolization, device thrombus, vascular access site complications, all bleeding events (including major bleeding), iatrogenic atrial septal defects (iASDs), and peridevice leaks. Secondary outcomes included procedural characteristics such as procedural time, fluoroscopy time, and the volume of contrast agent used.

### Statistical analysis

A meta-analysis was undertaken to assess variations between guidance provided by ICE and TEE concerning procedure-related parameters, procedural and non-procedural adverse events, and device-related adverse events. Findings are reported as mean differences (MD), standardized mean differences (SMD), odds ratios (OR), risk ratios (RR), or logRR alongside their respective 95% confidence intervals (Cis), as appropriate. In cases of studies that have zero events in one or both groups, a continuity correction was applied (constant 0.5). The degree of heterogeneity among studies was measured using I². Effect sizes were combined using a random-effects model. Significant heterogeneity was deemed present if I² values exceeded 50%. In cases of significant between-study heterogeneity, we conducted updated meta-analysis after removal of outlying studies. To explore potential publication bias, a contour-enhanced funnel plot was generated, and Egger’s test was conducted. Significance was defined by P values less than 0.05. All meta-analyses were generated using the meta and dmetar packages in R studio v.2023.12.1 + 402.

## Results

### Search results

**Fig. 1:**
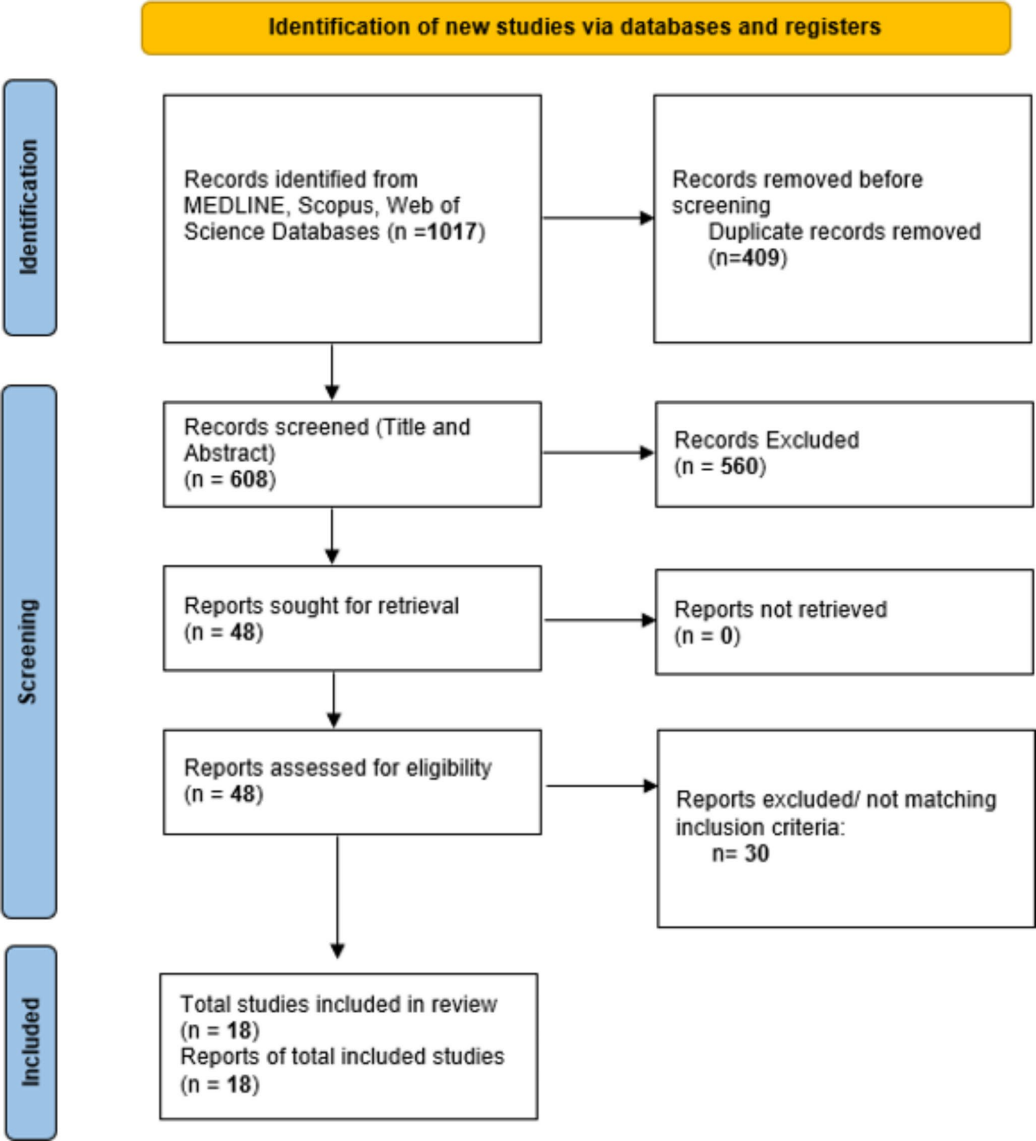
Flowchart of the processing of the included studies

The search yielded initially 1017 records. After removing the duplicates, a total of 608 studies were identified. Next, 560 studies were excluded based on their titles and abstracts and screening of the full texts of the remaining 48 articles resulted in the identification of 18 studies that met all eligibility criteria, as summarized in the PRISMA chart (Fig. 1).

### Study characteristics

This comparative meta-analysis of outcomes in ICE vs. TEE included 124,230 patients (ICE: 5278, TEE: 113297). Table 2 summarizes the study characteristics of the included studies. Follow-up among studies was variable and ranged from time to discharge up to 25 months. Nine studies were single center and eight were multicenter. The study conducted by Iwasawa et al. [25] was not characterized as single or multicenter (Table 2). Six studies were conducted in the USA [26–31], two in Italy [32, 33], two in Portugal [34, 35], one in China [36], one in Denmark [37], one in France [38], one in Korea [39], one in Poland [40], one in Switzerland [41], one was global [42], while one did not specify its location [25].

**Table 2:** Characteristics of the included studies in the meta-analysis

Study	*N*	Country	Type of study	Single vs. multicenter	Study period	LAAC device	Type of ICE catheter	ICE catheter position	Preprocedural imaging
Frangieh 2017 [26]	76	Switzerland	Prospective	single	november 2013-june 2016	watchman	AcuNav	LA	N/A
Iwasawa 2016 [27]	117	N/R	Retrospective	N/R	N/R	watchman	N/A	LA	N/A
Korsholm 2017 [28]	216	Denmark	Retrospective	Single	march 2010-november 2016	ACP / amulet	ViewFlex (st jude USA)	LA	Cardiac CT
Reis 2018 [29]	82	Portugal	Prospective	Single	May 2010 - January 2017	Watchman/ ACP / amulet	N/A	LA	TEE
Berti 2018 [30]	604	Italy	Retrospective	multicenter	December 2008 - April 2015	ACP / amulet	AcuNav	RA or LA	TEE and Cardiac CT
Kim 2018 [31]	144	Korea	Retrospective	Multicenter	March 2013- April 2017	Watchman/ ACP / amulet	AcuNav	LSPV	TEE
Streb 2019 [32]	23	Poland	Prospective	Single	NR	Amulet	AcuNav	LA	TEE
Hemam 2019 [33]	104	USA	Retrospective	multicenter	april 2015 - january 2018	watchman	AcuNav	LA	N/A
Nielsen-Kudsk 2019 [34]	1088	Global	Prospective	Multicenter	june 2015 - september 2016	amulet	ViewFlex *abbott	LSVP	TEE and Cardiac CT
Alkhouli 2020 [35]	286	USA	Prospective	Single	June 2016 - april 2019	watchman	AcuNavorViewFlex	LA	TEE and Cardiac CT
Grazina 2021 [36]	88	Portugal	Retrospective	Single	2009–2020	watchman / ACP / Amulet / Lambre	NR	LA	TEE and Cardiac CT
Gianni 2021 [37]	190	USA	NR	single	August - December 2020	watchman FLX	NR	LA	Not routinely applied
Pommier 2021 [38]	224	France	Prospective	single	january 2014 - april 2019	Watchman / ACP	ViewFlex Xtra	LA	Cardiac CT
Zahid 2022 [39]	61,995	USA	Retrospective	Multicenter	Quarter 4 of 2015 to 2019	NR	NR	NR	NR
Morcos 2022 [40]	18,448	USA	Retrospective	multicenter	2016–2018	NR	NR	NR	NR
Ferro 2023 [41]	39,759	USA	Prospective	multicenter	August 2020 - september 2021	Watchman FLX	NR	NR	TEE and Cardiac CT
Chu 2020 [42]	14	China	Prospective	Single	april -june 2019	Lambre	SoundStar, Biosense Webster, Diamond Bar, USA	LSPV	NA
Pastormerlo 2023 [43]	772	Italy	Prospective	multicenter	October 2018 - September 2021	watchman FLX	NR	LA	TEE or Cardiac CT

Abbreviations: ACP = Amplatzer Cardiac Plug, CT = Computed Tomography, ICE = Intracardiac Echocardiography, LA = Left Atrium, RA = Right Atrium, LSPV = Left Superior Pulmonary Vein, TEE = Transesophageal Echocardiography, USA = United States of America, NR = No Referred, NA = No Applied

Eleven studies included Watchman, four included the ACP/Amulet device, four studies included both and two studies included the LAmbre device (Table 2). In two studies, pre-procedural imaging involved either TEE or CCTA, while six studies utilized both imaging modalities. Notably, the studies conducted by Frangieh et al. [41], Iwasawa et al. [25], Hemam et al. [26], Zahid et al. [29], Morcos et al. [30], and Chu et al. [36] did not provide information on preprocedural imaging, as indicated in Table 2. In all included studies, patients in the TEE-guided group received general anesthesia or sedation, while those in the ICE-guided group did not.

Table 3 summarizes the main baseline characteristics of the patients in the included studies, The mean age of the patients included in the studies ranged from 58 to 88 years. There was no significant difference in mean age (ICE group: 74.7 ± 2.17 years, TEE group: 75.3 ± 2.02 years, p-value: 0.45) or CHA_2_DS_2_VASC score (ICE group: 4.43 ± 0.35, TEE group: 4.45 ± 0.338, p-value: 0.73) between TEE and ICE groups.

**Table 3:** Baseline characteristics of the patient population in the included studies

Study		Age	Gender (male)	LVEF	Mean CHAD2S-VASc	Mean HASBLED	HTN	DM	Prior Bleeding	History of stroke/TIA
Frangieh 2017 [26]	ICE (*n* = 32)	76 [68–80]	26(81%)	55 [42–61]	4 [3–5.8]	3 [3–4]	27(84%)	14(44%)	19(59%)	9(28%
	TEE (*n* = 44)	81 [75–85]	25(57%)	60 [55–63]	4 [3–5]	3 [3–4.8]	38(86%)	16(36%)	14(32%)	9(21%)
Iwasawa 2016 [27]	ICE (*n* = 22)	NR	NR	NR	4.7 ± 1.6	2.6 ± 1.2	NR	NR	NR	NR
	TEE (*n* = 95)	NR	NR	NR	4.4 ± 1.4	2.4 ± 1.0	NR	NR	NR	NR
Korsholm 2017 [28]	ICE (*n* = 109)	73.0 ± 7.8	68(62%)	60 (50–60)	4.1+/- 1.6	4.1 +/-0.9	91(84%)	23(21%)	94(86%)	50(46%)
	TEE (*n* = 107)	73.0 ± 9.7	79(74%)	60 (55–60)	4.4 +/-1.6	4.1 +/-1.1	86(80%)	23(22%)	86(80%)	59(55%)
Reis 2018 [29]	ICE (*n* = 26)	NR	NR	NR	NR	NR	NR	NR	NR	NR
	TEE (*n* = 56)	NR	NR	NR	NR	NR	NR	NR	NR	NR
Berti 2018 [30]	ICE (*n* = 187)	76 ± 8	153(82%)	53+/- 9	4.27 +/-1.40	3.25+/- 1.00	NR	NR	NR	NR
	TEE (*n* = 417)	74 ± 7	382(92%)	52+/- 11	4.25 +/-1.40	3.15 +/-1.10	NR	NR	NR	NR
Kim 2018 [31]	ICE (*n* = 41)	71.4 ± 9.3	24(59%)	18(44%)-EF < 40%	4.3+/-1.4	3.0+/-1.5	37(90%)	11(27%)	20(49%)	20(49%)
	TEE (*n* = 103)	72.3 ± 9.2	51(50%)	41(40%)-EF < 40%	4.3+/-1.4	3.1+/-1.4	86(84%)	26(25%)	45(44%)	44(43%)
Streb 2019 [32]	ICE (*n* = 11)	77[7]	5(46%)	55[14]	5[2]	3[1]	9(82%)	3(27%)	9(82%)	5(45%)
	TEE (*n* = 12)	73[15]	4(33%)	53.5[16.5]	5[1.5]	2[0.5]	11(92%)	3(25%)	10(83%)	3(25%)
Hemam 2019 [33]	ICE (*n* = 53)	77 ± 10	33(62%)	NR	4.5+/-1.8	NR	43(81%)	18(34%)	NR	22(42%)
	TEE (*n* = 51)	76 ± 7	31(61%)	NR	4.5+/-1.6	NR	46(90%)	15(29%)	NR	17(33%)
Nielsen-Kudsk 2019 [34]	ICE (*n* = 130)	75 ± 8	78(60%)	NR	4.1+/-1.6	3.2+/-0.9	NR	NR	94(72%)	70(54%)
	TEE (*n* = 955)	75 ± 9	621(65%)	NR	4.2+/-1.6	3.3+/-1.1	NR	NR	688(72%)	334(35%)
Alkhouli 2020 [35]	ICE (*n* = 90)	75.7 ± 8.0	56(62%)	55.3+/-11.6	4.7+/-1.4	2.8+/-1.2	83(92%)	30(33%)	NR	33(36%)
	TEE (*n* = 196)	75.2 ± 7.8	109(56%)	58.0+/-9.1	4.8+/- 1.6	2.9+/-1.1	171(87%)	86(44%)	NR	84(43%)
Grazina 2021 [36]	ICE (*n* = 45)	75.9 ± 10.3	30 (67%)	NR	3.96 +/- 1.43	3.62 +/- 1.11	31 (69%)	15(33%)	34 (76%)	NR
	TEE (*n* = 43)	74.2 ± 9.7	28(65%)	NR	4.07 +/- 1.35	3.63 +/- 1.00	36(84%)	13(30%)	30(70%)	NR
Gianni 2021 [37]	ICE (*n* = 122)	72 ± 8	81(66%)	NR	4.1 ± 1.4	2.7 ± 1.3	NR	NR	NR	NR
	TEE (*n* = 68)	75 ± 9	41(60%)	NR	4.3 ± 1.3	2.7 ± 1.2	NR	NR	NR	NR
Pommier 2021 [38]	ICE (*n* = 175)	76 ± 8	122(70%)	57 ± 7	4.2 ± 1.4	4.1 ± 1.0	160(91%)	60(34%)	161(91%)	122(70%)
	TEE (*n* = 49)	75 ± 7	35(71%)	57 ± 7	4.5 ± 1.5	3.9 ± 1.0	46(94%)	10(20%)	41(84%)	37(77%)
Zahid 2022 [39]	ICE (*n* = 1410)	75 (69–79)	870 (62%)	NR	NR	NR	1215(86%)	290(21%)	NR	NR
	TEE (*n* = 60585)	77 (71–82)	35,315 (58%)	NR	NR	NR	52,575(87%)	11,370(19%)	NR	NR
Morcos 2022 [40]	ICE (*n* = 397)	70.7 ± 0.7	235(59%)	NR	NR	NR	236 (60%)	68 (17%)	NR	NR
	TEE (*n* = 18051)	75.3 ± 1.4	10,838(60%)	NR	NR	NR	10,308 (57%)	3391(19% )	NR	NR
Ferro 2023 [41]	ICE (*n* = 2272)	75.8 ± 8.0	1365 (60%)	54.3+/- 10.2	4.8+/- 1.5	2.5+/- 1.0	2083(92%)	837(37%)	NR	524(23%)
	TEE (*n* = 31835)	76.4 ± 7.9	18,817 (59%)	54.0 +/- 9.9	4.8+/- 1.5	2.4+/- 1.0	29,194(92%)	1350(4%)	NR	6851(22%)
Chu 2020 [42]	ICE (*n* = 7)	71.7 ± 8.8	5(71%)	60.9 ± 10.6	5.1 ± 2.1	3.0 ± 1.2	5 (71%)	0(0%)	1(14%)	6(86%)
	TEE (*n* = 7)	75.6 ± 9.1	4(57%)	64.3 ± 5.4	5.1 ± 1.2	3.1 ± 0.7	6(86%)	1(14%)	1(14%)	4(57%)
Pastormerlo 2023 [43]	ICE (*n* = 149)	77 ± 7.5	97(65%)	54 ± 11	4.2 ± 1.8	3.5 ± 1.4	115(77%)	45(30%)	40 (27%)	19(13%)
	TEE (*n* = 623)	76.3 ± 8	407(65%)	51 ± 11	4.1 ± 1.4	3.7 ± 1.1	491(79%)	219(35%)	200(32%)	87(14%)

### Primary outcome

#### Primary efficacy outcome

##### **Technical success**

**Fig. 2:**
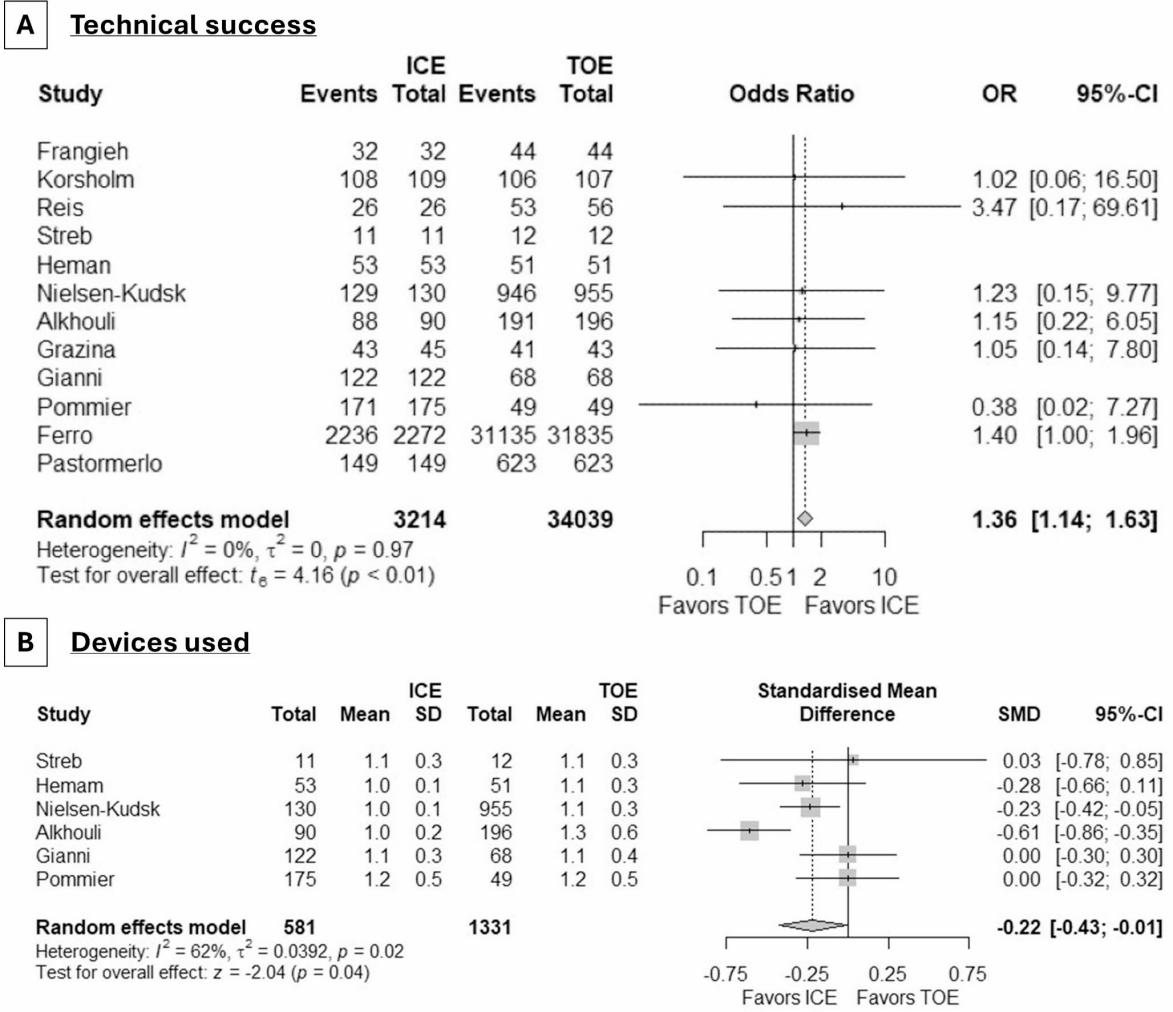
Forest plot demonstrating the differences in **(A)** technical success and **(B)** devices used between ICE and TEE in LAAC procedures. OR: odds ratio, CI: confidence interval, SMD: standardized mean difference

We analyzed a total of 12 studies with 37,253 patients (ICE: 3214, TEE: 34039) for differences in technical success, with higher odds with the use of ICE (OR: 1.36, 95% CI: 1.14, 1.63, *p* = 0.006) (Fig. 2, **Panel A**). No evidence of between-study heterogeneity (I^2^ = 0%) was present. Concerning the number of devices used, a meta-analysis of 6 studies indicated that significantly fewer were employed in cases of ICE guidance (SMD: -0.22, 95% CI: -0.43, -0.01, *p* = 0.04, I^2^ = 62%) (Fig. 2, **Panel B**), without any outlying studies.

#### Primary safety outcome

##### Procedure-related complications

**Fig. 3:**
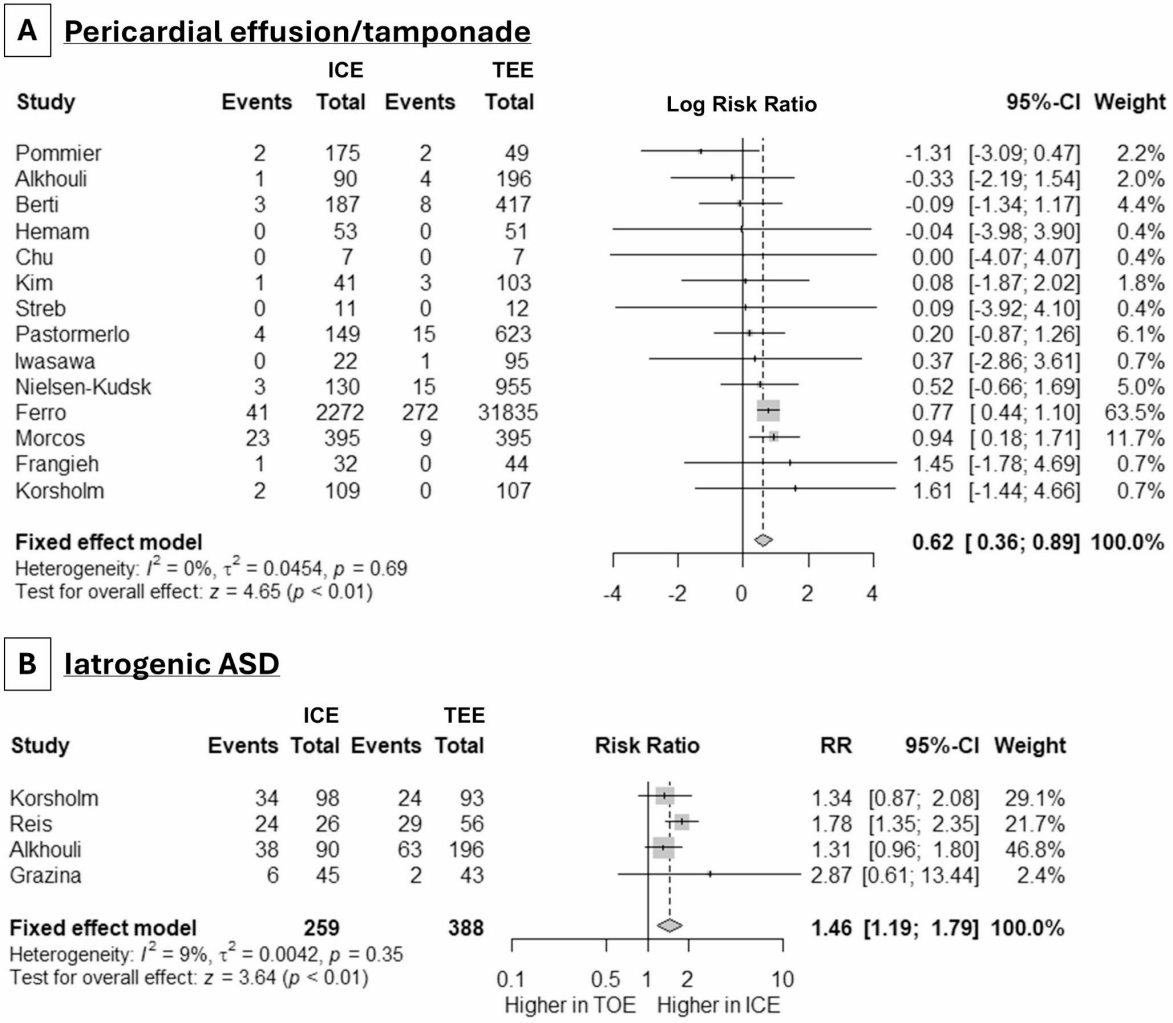
Forest plot demonstrating the differences in the incidence of **(A)** pericardial effusion/tamponade and **(B)** iatrogenic atrial septal defect (ASD) between ICE and TEE in LAAC procedures. RR: risk ratio, CI: confidence interval

**Fig. 4:**
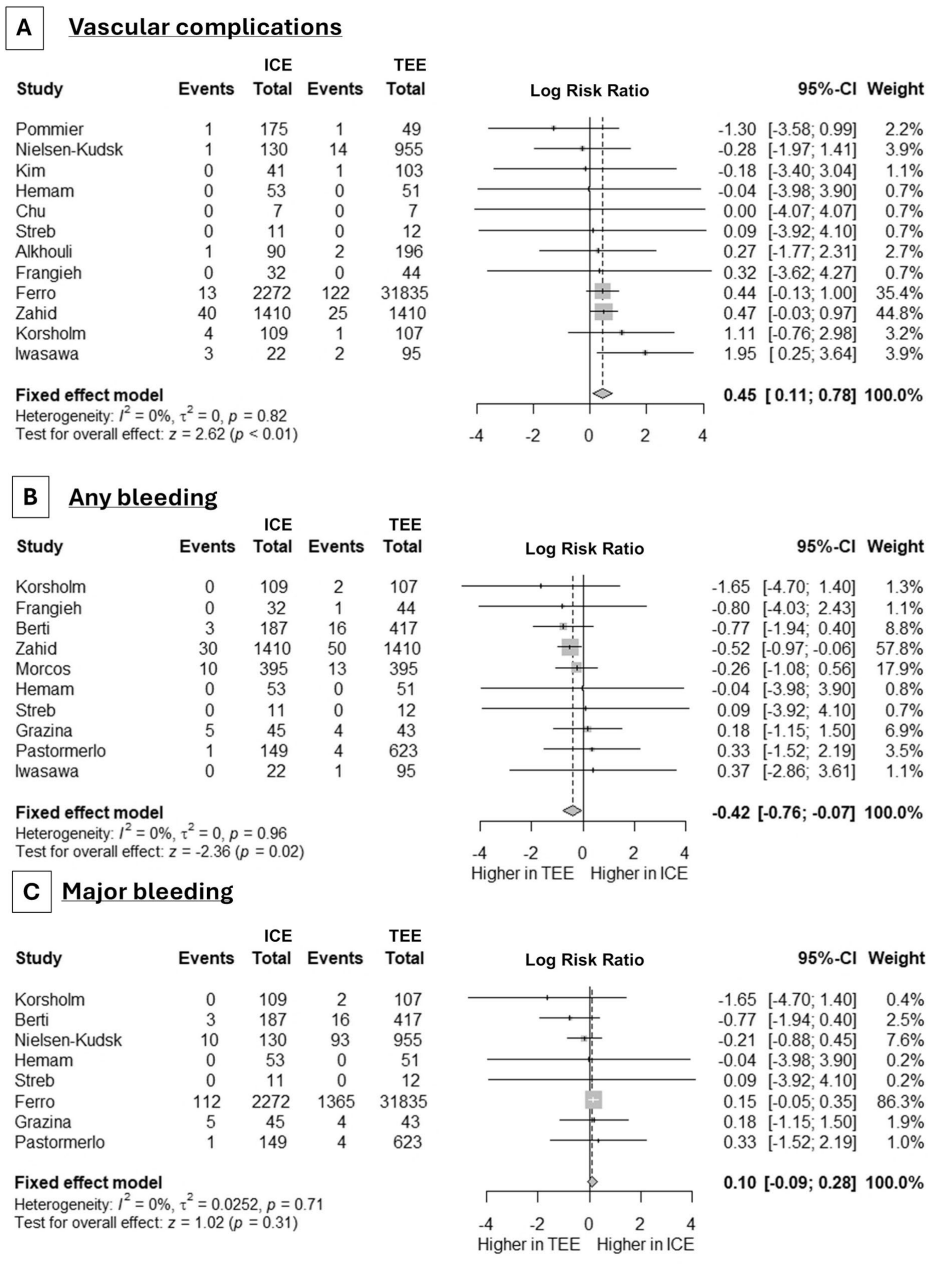
Forest plot demonstrating the differences in the incidence of **(A)** vascular complications, **(B)** any bleeding, and **(C)** major bleeding events between ICE and TEE in LAAC procedures. RR: risk ratio, CI: confidence interval

We proceeded to the assessment of LAAO safety by the evaluation of procedure- or device-related adverse events. While there was no difference in procedure- or device-related adverse events in the studies that assessed this combined endpoint (5 studies, RR: 0.98, 95% CI: 0.68, 1.41, *p* = 0.86, I^2^ = 0%), the use of ICE was accompanied by a greater risk of pericardial effusion/tamponade (11 studies, logRR: 0.62, 95% CI: 0.36, 0.89, *p* < 0.001) (Fig. 3, **Panel A**), with no between-study heterogeneity (I^2^ = 0%). As far as iASDs are concerned, we noted a greater incidence in cases of ICE use (4 studies, RR: 1.53, 95% CI: 1.12, 2.09, *p* = 0.02, I^2^ = 1%) (Fig. 3, **Panel B**). More vascular complications were noted in cases of ICE (logRR: 0.45, 95% CI: 0.11, 0.78, *p* = 0.009) (Fig. 4, **Panel A**). Interestingly, any bleeding events were met less frequently in cases of ICE use (8 studies, logRR: -0.42, 95% CI: -0.76, -0.07, *p* = 0.02, I^2^ = 0%) (Fig. 4, **Panel B**), but major bleeding was equally observed across the two methods (6 studies, logRR: 0.10, 95% CI: -0.09, 0.28, *p* = 0.31, I^2^ = 0%) (Fig. 4, **Panel C**).

#### Device-related complications

**Fig. 5:**
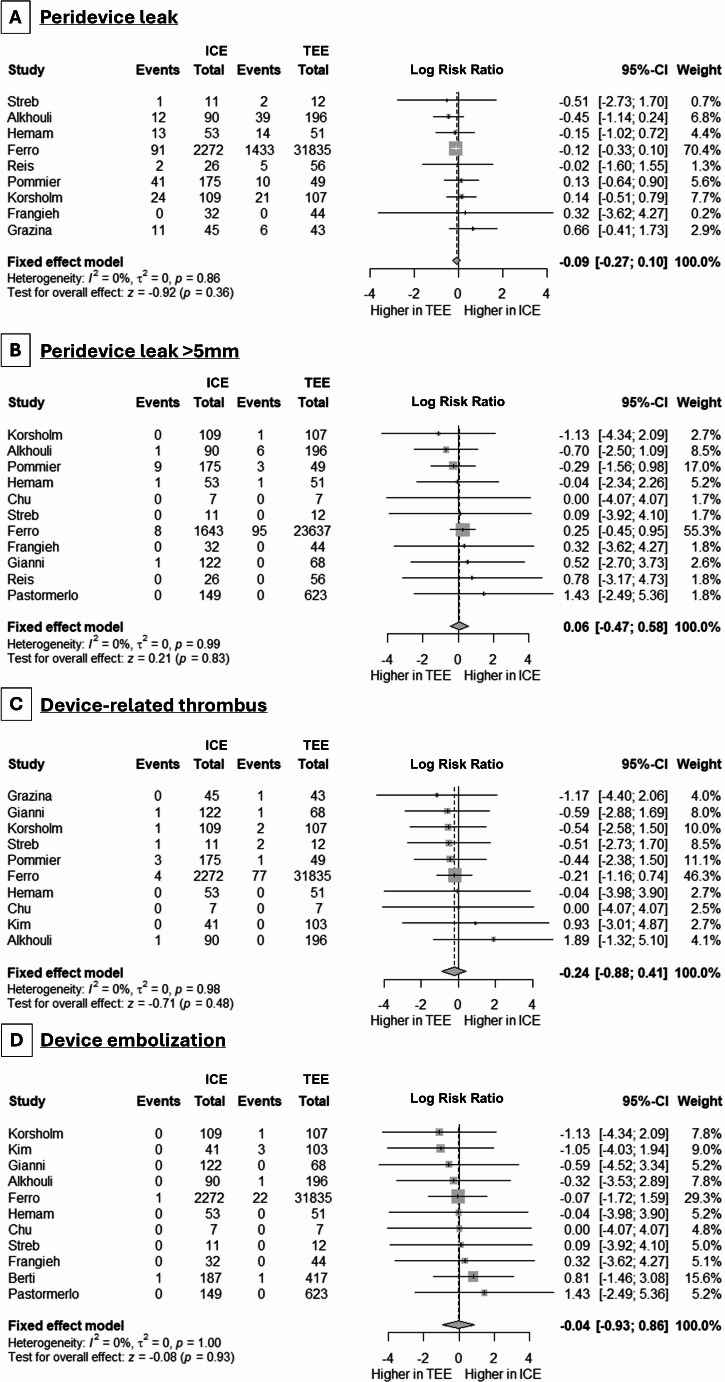
Forest plot demonstrating the differences in the incidence of **(A)** peridevice leak, **(B)** peridevice leak > 5 mm, **(C)** device-related thrombus, and **(D)** device embolization between ICE and TEE in LAAC procedures. RR: risk ratio, CI: confidence interval

The presence of any PDL was equally noted when using ICE and TEE (8 studies, logRR: -0.09, 95% CI: -0.27, 0.10, *p* = 0.36, I^2^ = 0%) (Fig. 5, **Panel A**), with similar findings in cases of significant (> 5 mm) PDL (6 studies, logRR: 0.06, 95% CI: -0.47, 0.58, *p* = 0.83, I^2^ = 0%) (Fig. 5, **Panel B**). Regarding device-related thrombus, its incidence was less frequent -albeit statistically nonsignificant- in cases of ICE use (7 studies, logRR: -0.24, 95% CI: -0.88, 0.41, *p* = 0.48, I^2^ = 0%) (Fig. 5, **Panel C**). Device embolization was infrequent and was noted similarly in cases of ICE and TEE use (5 studies, logRR: -0.04, 95% CI: -0.93, 0.86, *p* = 0.93, I^2^ = 0%) (Fig. 5, **Panel D**).

### Secondary outcomes

#### Procedure-related parameters

**Fig. 6:**
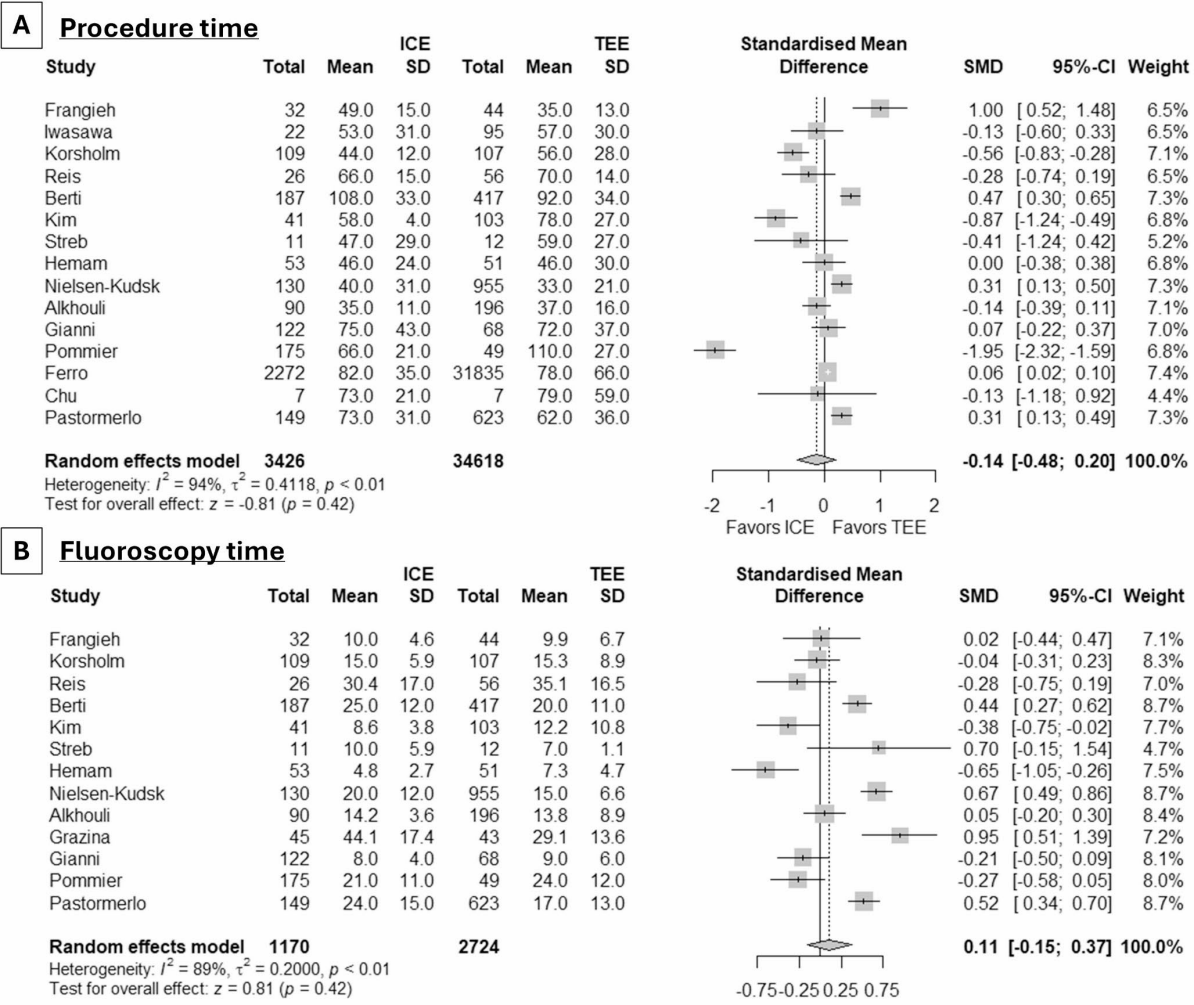
Forest plot demonstrating the differences in **(A)** procedure time and **(B)** fluoroscopy time between ICE and TEE in LAAC procedures. MD: mean difference, CI: confidence interval

We also assessed whether the use of different imaging modalities had any effect on procedure or fluoroscopy time. The meta-analysis of 15 studies (38044 patients) showed no difference between ICE and TEE in procedure time (SMD: -0.14, 95% CI: -0.48, 0.2, *p* = 0.42) (Fig. 6, **Panel A**). Due to the significant between-study heterogeneity (I^2^: 94%), we conducted an updated meta-analysis after removal of outlying studies (Frangieh et al., Berti et al., Kim et al., and Pommier et al.), with similar findings to the original analysis (SMD: -0.03, 95% CI: -0.20, 0.15, *p* = 0.77, I^2^ = 76%). The meta-analysis of 13 studies (3894 patients) did not detect significant variations in fluoroscopy time (SMD: 0.11, 95% CI: -0.15, 0.37, *p* = 0.42) (Fig. 6, **Panel B**), while the updated meta-analysis after removal of outlying studies (Grazina et al., Hemam et al., and Nielsen-Kudsk et al.) produced similar observations (SMD: 0.04, 95% CI: -0.18, 0.27, *p* = 0.72, I^2^ = 84%). Contrast medium volume did not differ according to the use of ICE or TEE in the original (SMD: 0.02, 95% CI: -0.23, 0.26, *p* = 0.89, I^2^: 87%) and updated meta-analysis (SMD: 0.01, 95% CI: -0.22, 0.24, *p* = 0.93, I^2^: 76%).

### Publication bias

The egger’s test on the predetermined outcomes (displayed in Supplementary Table 9) yielded nonsignificant results. In line with these observations, inspection of contour-enhanced funnel plots did not reveal asymmetry, confirming an overall low risk of publication bias (Supplementary Figs. 2–8).

## Discussion

### Main findings

This meta-analysis has shown the following key findings:1) ICE-guided LAAO is associated with higher technical success and a significantly lower mean number of devices used compared to TEE-guided LAAO; 2)No significant differences were observed in total procedural or fluoroscopy time, as well as in contrast medium volume used; 3) A greater incidence of pericardial effusion/tamponade, iASDs and vascular complications were observed in ICE-guided group and 4) No significant differences were noted in device-related adverse events between the ICE- guided and the TEE- guided groups.

Our extensive literature search identified 9 meta-analyses and 4 systematic reviews that demonstrated similar technical success rates with both two imaging modalities without an increase in risk of complications [43–52].Compared with previous meta-analyses, we included more studies due to the search strategy and eligibility criteria and one additional study (Ferro et al., 2023^41^) may have increased the power to estimate an actual difference between the two imaging modalities as it included a larger number of patients [31].

### Clinical implications

#### Technical success / devices used

In our meta-analysis, technical success showed higher odds with the use of ICE with a lower mean number of device attempts in cases of ICE guidance. This comes in contrast to commonly reported concerns that ICE imaging may provide inferior imaging to guide device placement leading to suboptimal closure [53, 54]. Compared to TEE, ICE allows the operator to directly control the imaging guidance. As a result, a more successful device implantation is achieved even though TEE allows imaging to be guided by another operator enabling them to perform adequate imaging more efficiently. Moreover, ICE in left atrium (LA) position can be utilized in deploying LAAO devices with similar efficacy as TEE as it can provide more clear and detailed imaging of the structure of the LAA due to higher image resolution. However, other factors, including specific anatomical characteristics of the LAA, may influence the safe release of the device. For instance, Korsholm et al. [37] reported a case in the ICE group where a shallow LAA neck, previously identified in the preprocedural CT, resulted in unsuccessful device implantation. Similarly, Reis [34] and Kim [39] noted that technical failures occurred when the LAA was either too small or too large, preventing effective sealing, respectively. Moreover, in studies where complete technical success was not achieved, various factors unrelated to the imaging modality were identified as causes of unsuccessful device implantation. These included technique-related issues, such as a loose connection between the device and the delivery cable [27], as well as the presence of severe venous disease [34].

The reliability of LA ICE for device sizing is also questionable. However, ICE measurements from the right atrium or coronary sinus position correlate well with results from fluoroscopy for LAA sizing and the combination of these measurements offers the operator the ability to accurately choose the appropriate device size [55].Considering the inherent limitation to size LAA by ICE due to the lack of 3-dimensional capabilities and the high eccentricity of the LAA, patients receiving computed tomography (CT) scan before procedure were found to have less frequent need for a second device in the multicenter study conducted by Berti et al. [55]. Preprocedural CT imaging has been shown to increase both specificity and positive predictive value without compromising sensitivity for LAAO procedural planning [56, 57].

#### Pericardial effusion/tamponade

A significantly higher incidence of pericardial effusion or tamponade in the ICE-guided group was found in our analysis. This was not a surprising finding and is consistent with prior reports [32, 58–61] and may be justified by the fact that ICE requires more catheter manipulation to cross the interatrial septum and obtain the target views, increasing the procedural risk. Piercing the LA or aortic root during transeptal puncture, piercing LA or LAA due to improper catheter manipulation, and tearing the LAA during the device deployment or retraction are among the leading causes of pericardial effusion [62].

Unlike ICE-guided atrial septal closure wherein septal interrogation is performed primarily in the right atrium with little manipulation, LAAO may require maneuvering of the ICE catheter across the interatrial septum within the LA, or within the coronary sinus or left pulmonary artery. Moreover, limited operator experience with LA ICE may also have contributed to the observed differences in pericardial effusion events as a learning curve is required in order to acquire proficiency in LAAO guidance with ICE. Concomitant use of ICE and TEE as operators gain experience with ICE, training and/or proctoring by physicians experienced with ICE-guided LAAO may also help to minimize the risk of complications during initial experiences with ICE-guided cases.

#### Iatrogenic ASDs

A higher prevalence of residual iASDs was also observed with ICE-guided LAAO. This can be explained by both single- and double- puncture techniques used for ICE catheter advancement into the LA, pulmonary artery, or the left superior pulmonary vein [63, 64]. In most studies, a single transeptal puncture was performed and the ICE catheter and the delivery sheath were inserted through the same puncture, further dilating the puncture site in order to facilitate the passage of the ICE catheter. Also, the additional probe manipulation of the 2-dimensional ICE that is frequently required in order to change views may further increase the size of transeptal hole. These factors may explain the higher risk of iASD seen with ICE in our analysis. Also, the majority of iASDs were depicted at the 45-day follow-up imaging. As most iASDs close spontaneously by 3–12 months, longer-term follow-up is required in order to establish the clinical impact of these residual shunts. Nonetheless, according to the literature on iASDs after AF ablation or transcatheter mitral procedures, their persistence does not result in increased risk of paradoxical embolism or other clinical manifestations [65, 66]. However, the incidence of residual iASDs was poorly investigated in the majority of the included studies, with a total of four studies assessing iASDS occurrence [27, 34, 35, 37]. Further randomized trials are still needed to confirm these findings by long- term follow‐up.

#### Vascular complications

We noticed an increased rate of vascular complications in the ICE-guided group. ICE guidance requires an additional venous vascular access site, thus increasing the rate of vascular complications and possibly leading to an increase in hospital stay and costs. Ultrasound-guided venous access and careful maneuvers can reduce access-related complications; however this has to be further tested in future trials.

#### Procedural, fluoroscopy times and contrast volume agents

Our meta-analysis showed similar procedural, fluoroscopy times and contrast volume consumption with both techniques. However, significant heterogeneity was evident across studies for these endpoints which may be related to the experience of the operators. Few studies have shown shorter fluoroscopy time, interventional procedure time, and catheterization laboratory time in ICE-guided group compared with TEE [67, 68]. Specifically, in the study conducted by Kim et al. [39], total procedure time was significantly lower in the ICE-guided LAAO group, pointing out that placing the ICE probe at the ostium of left superior pulmonary vein offers quite similar image to TEE view. These results are in contrast to a previous report by Frangieh et al. [41] which showed longer total procedure time in the ICE-guided group compared to TEE, with the learning-curve of the ICE contributing to prolonged procedure time. Similarly, Berti et al. [32] showed longer procedural and fluoroscopy time in the ICE-guided group. Unfortunately, no direct comparison can be performed due to the included heterogeneity of the learning curve of the operators and the different time intervals of the LAAO procedure used along the studies. As the level of the technique and experience is not consistent in each center, the amount of contrast usage is greatly different. Nielson-Kudsk et al. [42] found that ICE‐guided procedure takes longer fluoroscopy time and uses more contrast than TEE‐guided procedure. Hence, this would be an interesting point for future research as chronic kidney disease is common in the population targeted for LAAO [32, 34, 41, 60].

#### Clinical perspectives

Although expert consensus documents are starting to emerge, standardized ICE imaging protocols are needed to disseminate best practices that can improve both periprocedural effectiveness and safety. Also, preprocedural CT scans may help to facilitate navigation and improve safety, while the introduction of 4D ICE may markedly decrease the need for catheter manipulation in the LA. The availability of newer, softer catheters may further promote safety among operators as they become familiar with the ICE technology.

Newer TEE models (with 3D capabilities) are also introduced into the market al.lowing TEE to be more regularly conducted with deep sedation instead of general anesthesia in many centers [69]. Hence, the cost-effectiveness considerations of ICE and TEE will continue to evolve over time and require further study [22, 26, 29, 30].

### Limitations

The results of this study should be interpreted with caution in the context of the following limitations. The studies included in this meta-analysis were non-randomized and observational in design, which predisposes our analysis to selection or misclassification bias given the variability in inclusion and exclusion criteria of individual studies. Furthermore, this is a study-level meta-analysis, and therefore should be interpreted under its limitations. Additionally, this meta-analysis includes studies with mostly small sample sizes which may affect the stability of the result indicators, reduce the efficiency of the test, and introduce potential research bias. We also included retrospective studies which may be subjected to confounding bias. Also, as most of the data of our analysis are derived from non-granular databases, evaluating the technical success rate is fairly challenging. Besides, due to the variability in post-procedural follow-up timing and the lack of sufficient data, analyzing the impact of device type and size on PDL proved difficult. Finally, the learning curve and health costs may have affected the use of ICE. Variability in studies is further complicated by operator experience which also contributes to selection bias towards TEE as ICE is a relatively novel technology. A prospective, multicenter, randomized, well-controlled study is needed to clarify the clinical outcomes of LAAO with the comparison of ICE vs. TEE monitoring.

## Conclusion

In conclusion, although TEE remains the gold standard imaging modality during LAAO, the present meta-analysis shows that ICE compared to TEE is associated with higher technical success and a significantly lower mean number of devices used. However, ICE was associated with a higher prevalence of residual iASDs, pericardial effusions and vascular complications. These results suggest that well-designed studies are currently needed to compare safety and efficacy of these imaging modalities during LAAO.

## Electronic supplementary material

Below is the link to the electronic supplementary material.

Supplementary Material 1

Supplementary Material 2

Supplementary Material 3

Supplementary Material 4

Supplementary Material 5

Supplementary Material 6

Supplementary Material 7

Supplementary Material 8

Supplementary Material 9

## Data Availability

No datasets were generated or analysed during the current study.

## Abbreviations

AF: Atrial fibrillation
LAA: Left atrial appendage
OAC: Oral anticoagulation
LAAO: Left atrial appendage occlusion
ACP: Amplatzer cardiac plug 1
CCTA: Cardiac computed tomography angiography 2
TEE: Transesophageal echocardiography
ICE: Intracardiac echocardiography
iASD: iatrogenic atrial septal defect
LA: Left atrium
CT: Computed tomography
