# Supplementary figures and images for: Intracardiac or transesophageal echocardiography for left atrial appendage occlusion: an updated systematic review and meta-analysis
Source: Int J Cardiovasc Imaging. 2025 Jan 22;41(3):489–505. doi: 10.1007/s10554-025-03330-z (PMC11880089; doi:10.1007/s10554-025-03330-z)

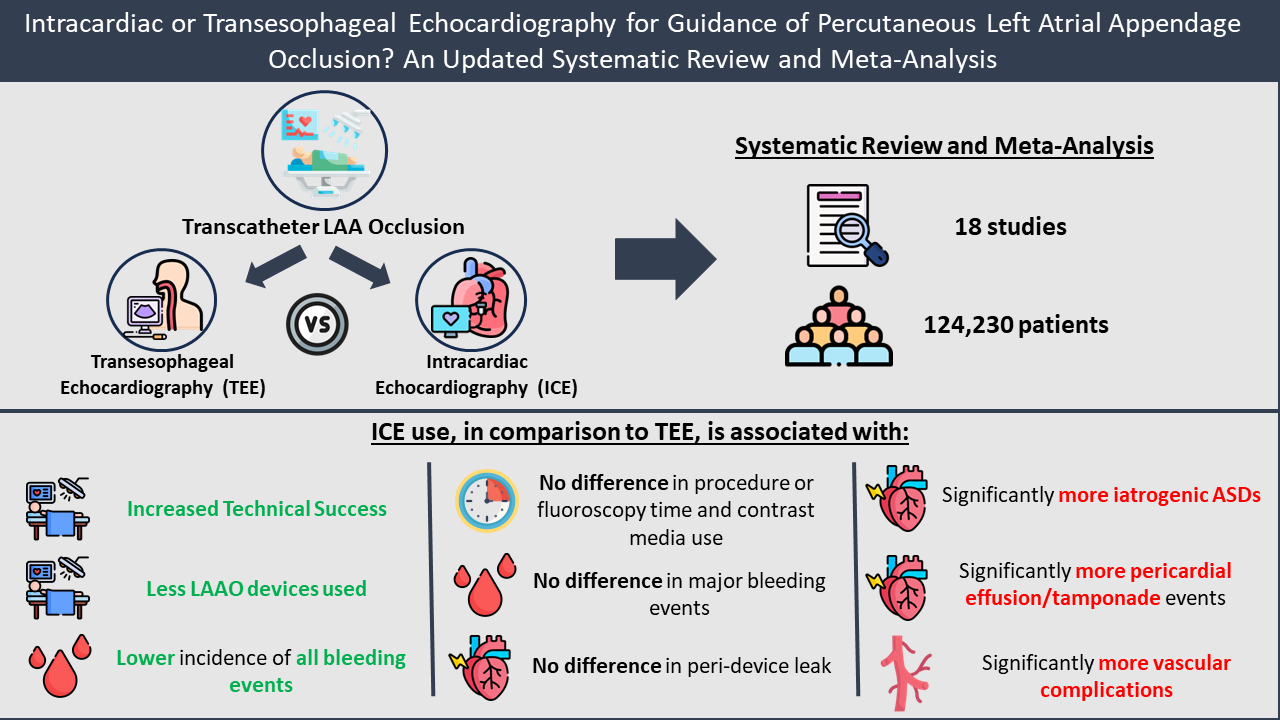

Supplement: Supplementary file 1 — Supplementary Material 1 [file 10554_2025_3330_MOESM1_ESM.tiff]

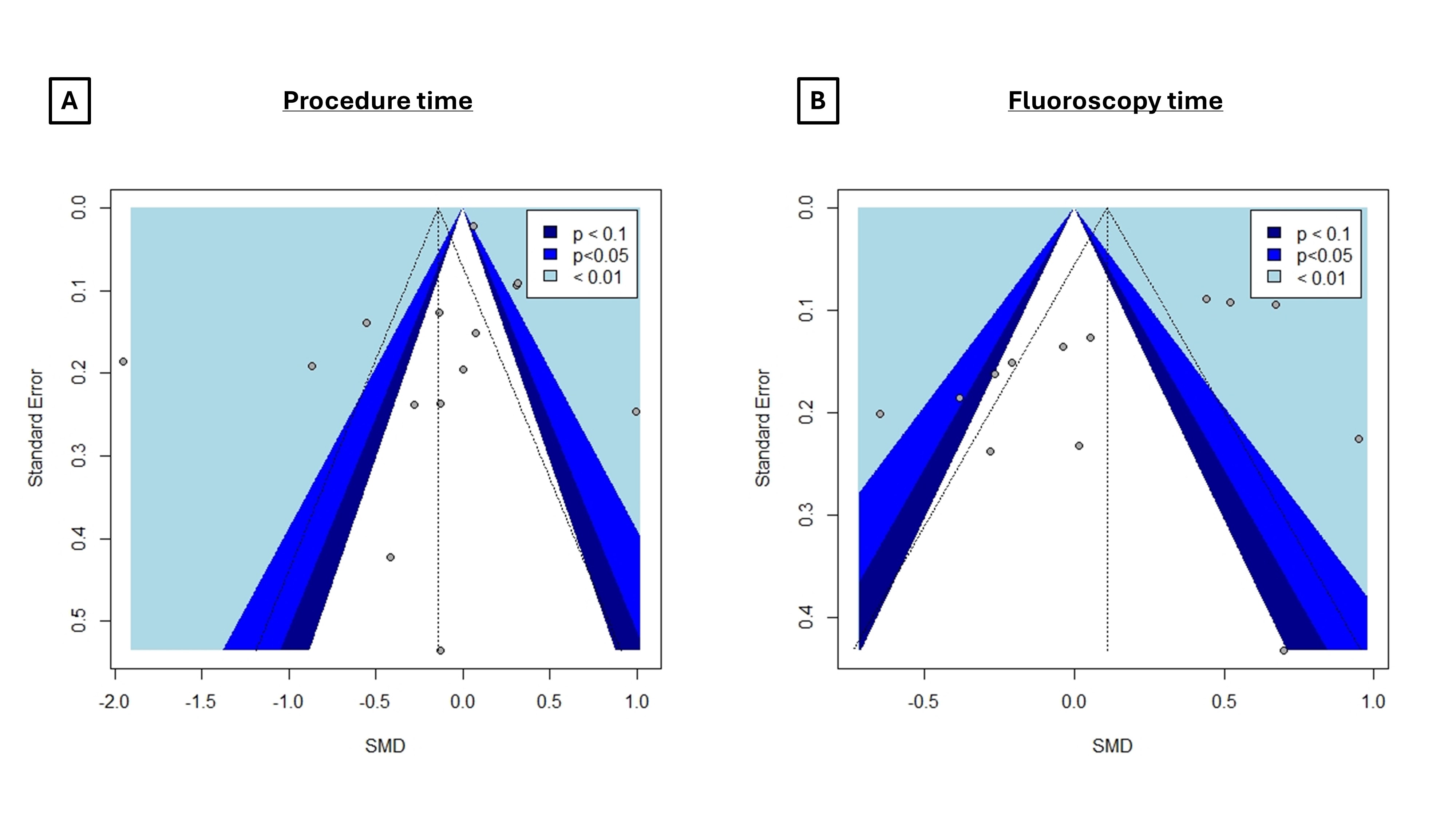

Supplement: Supplementary file 2 — Supplementary Material 2 [file 10554_2025_3330_MOESM2_ESM.jpg]

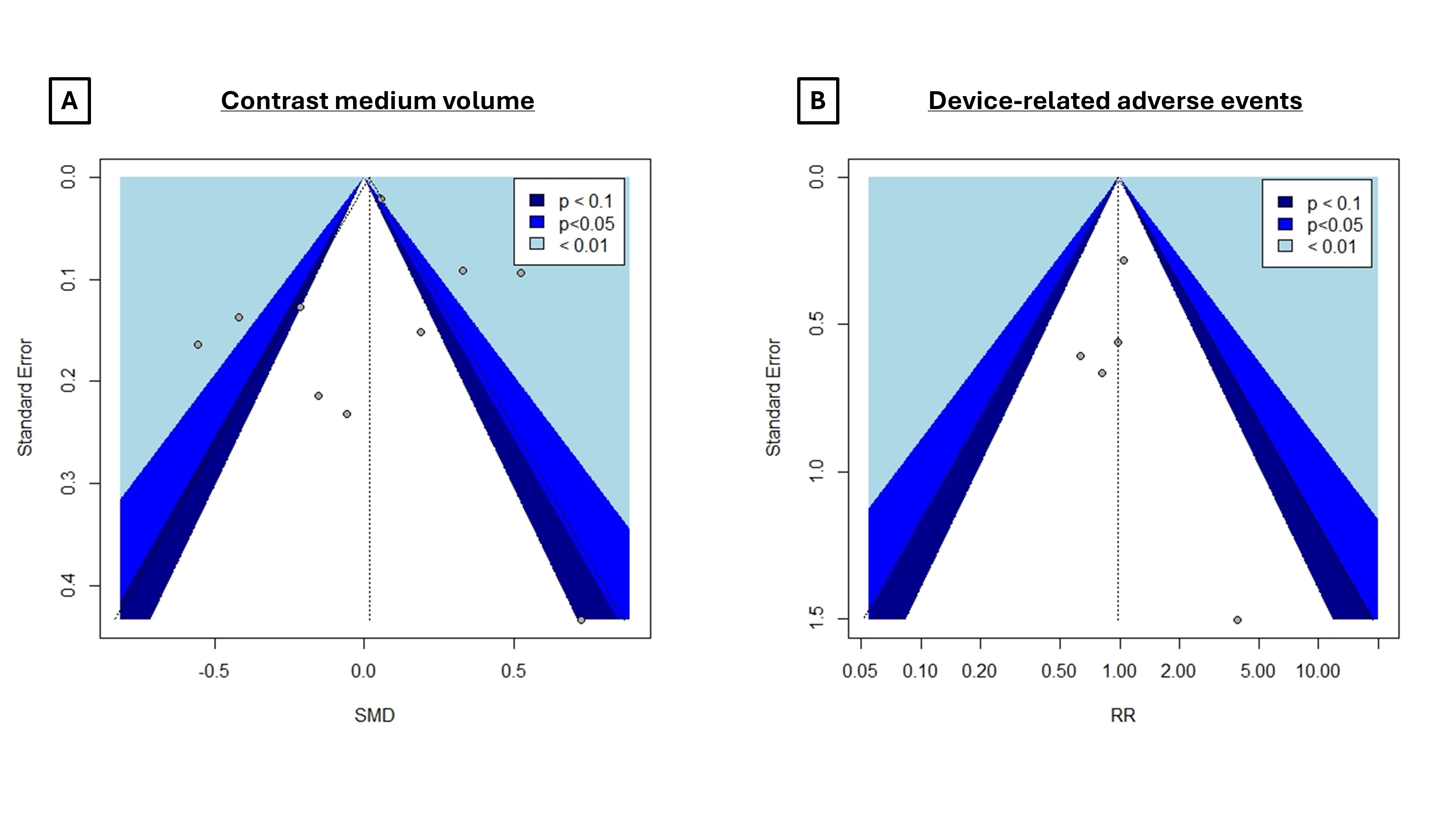

Supplement: Supplementary file 3 — Supplementary Material 3 [file 10554_2025_3330_MOESM3_ESM.jpg]

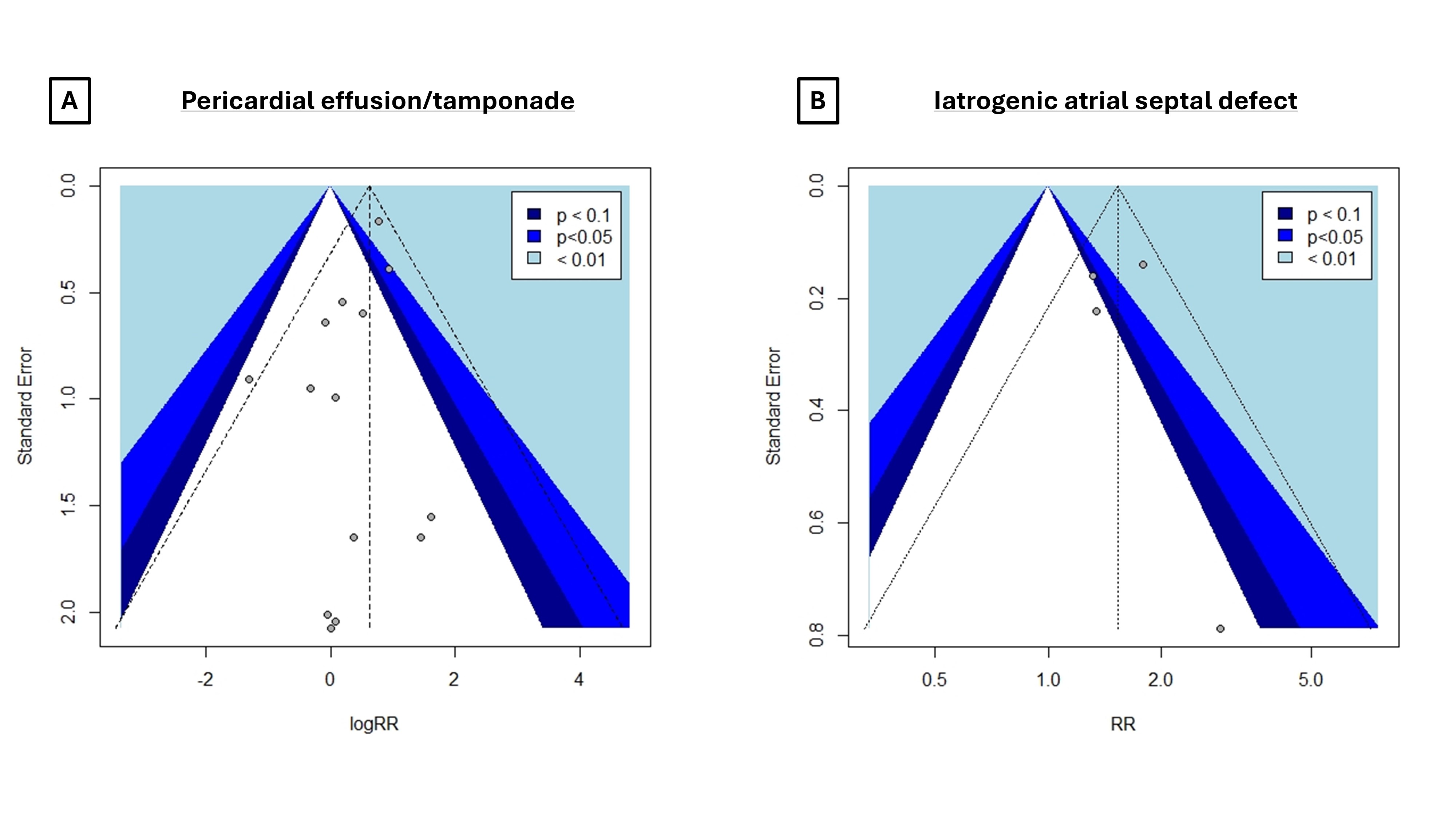

Supplement: Supplementary file 4 — Supplementary Material 4 [file 10554_2025_3330_MOESM4_ESM.jpg]

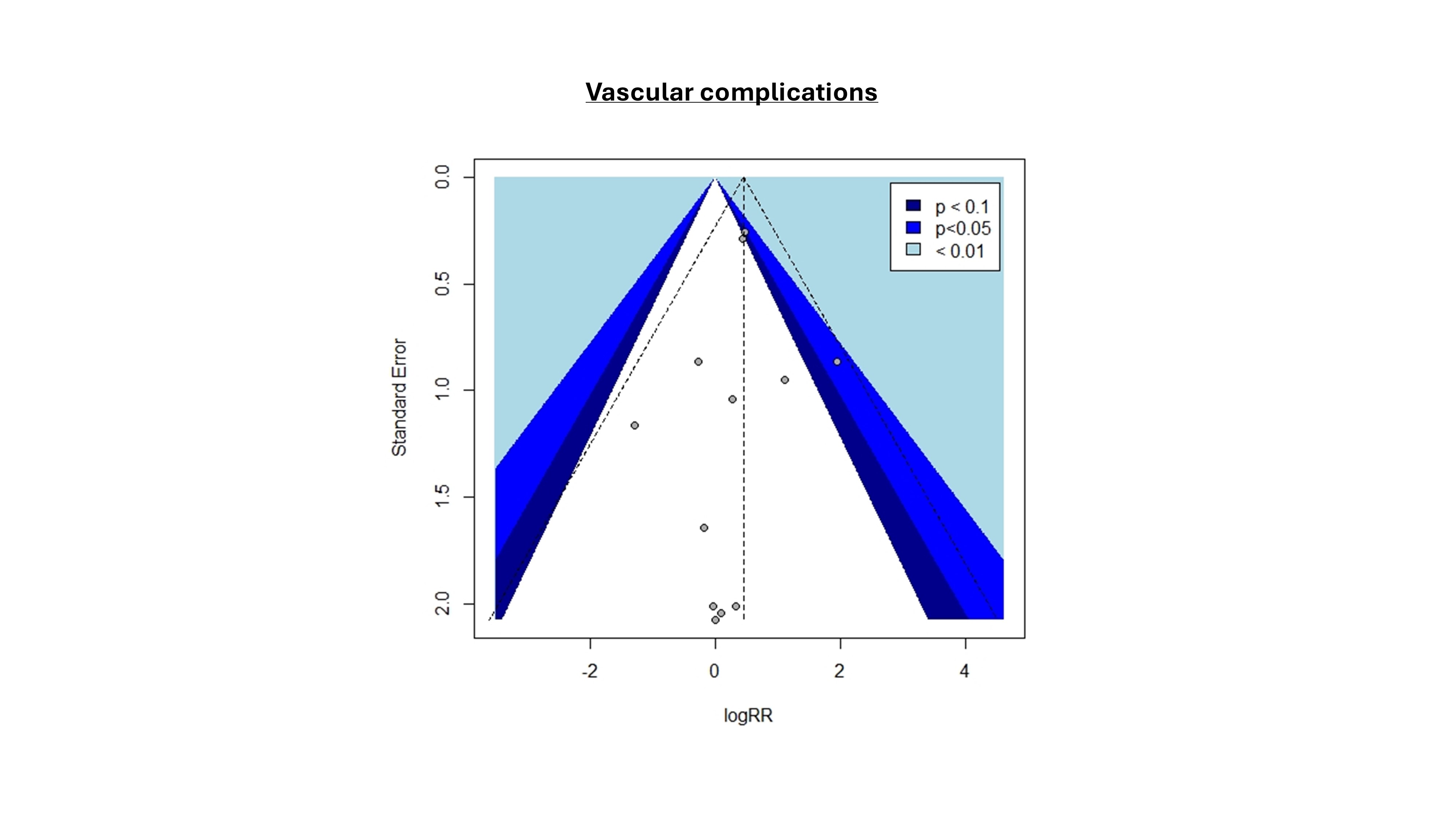

Supplement: Supplementary file 5 — Supplementary Material 5 [file 10554_2025_3330_MOESM5_ESM.jpg]

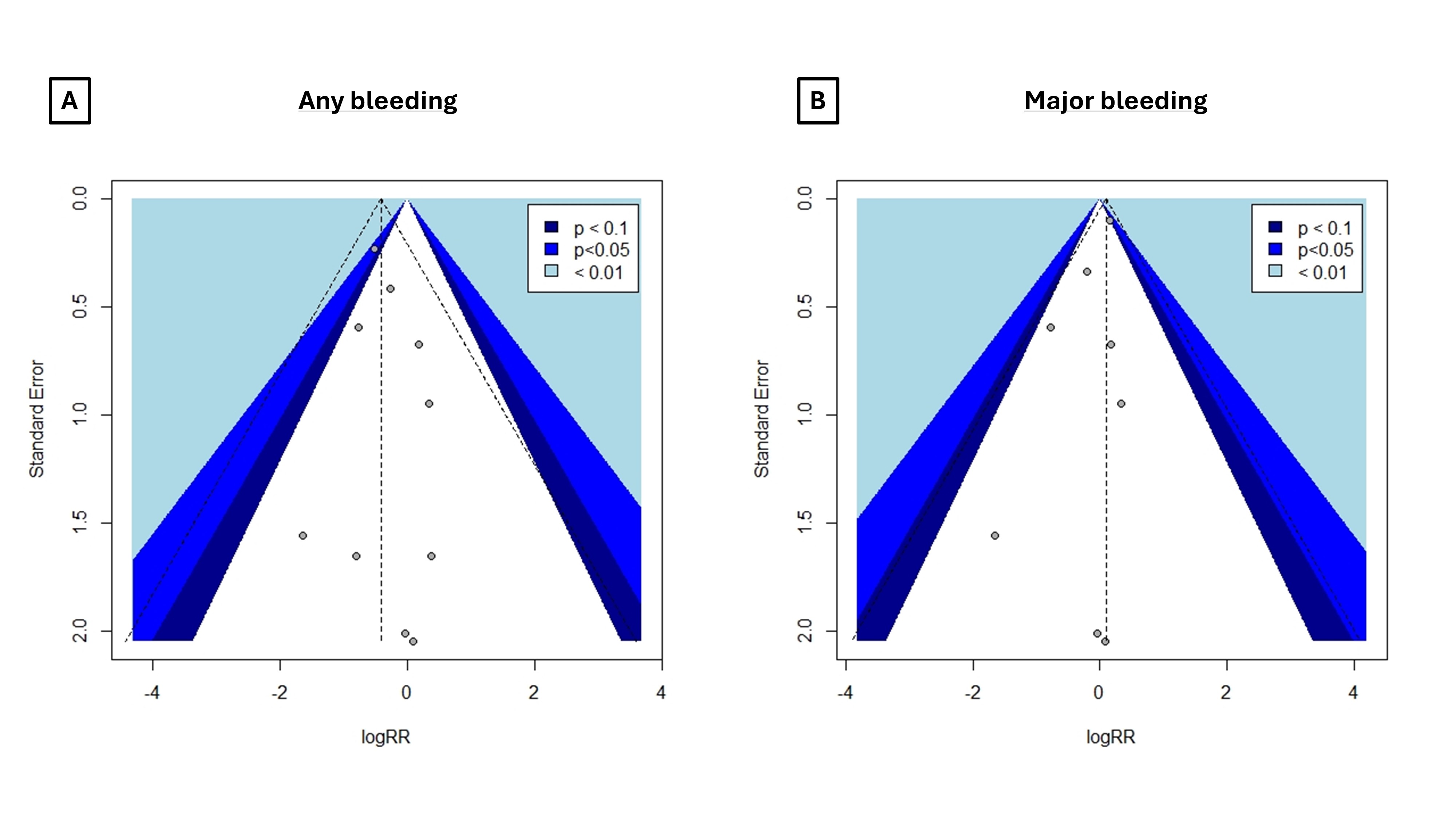

Supplement: Supplementary file 6 — Supplementary Material 6 [file 10554_2025_3330_MOESM6_ESM.jpg]

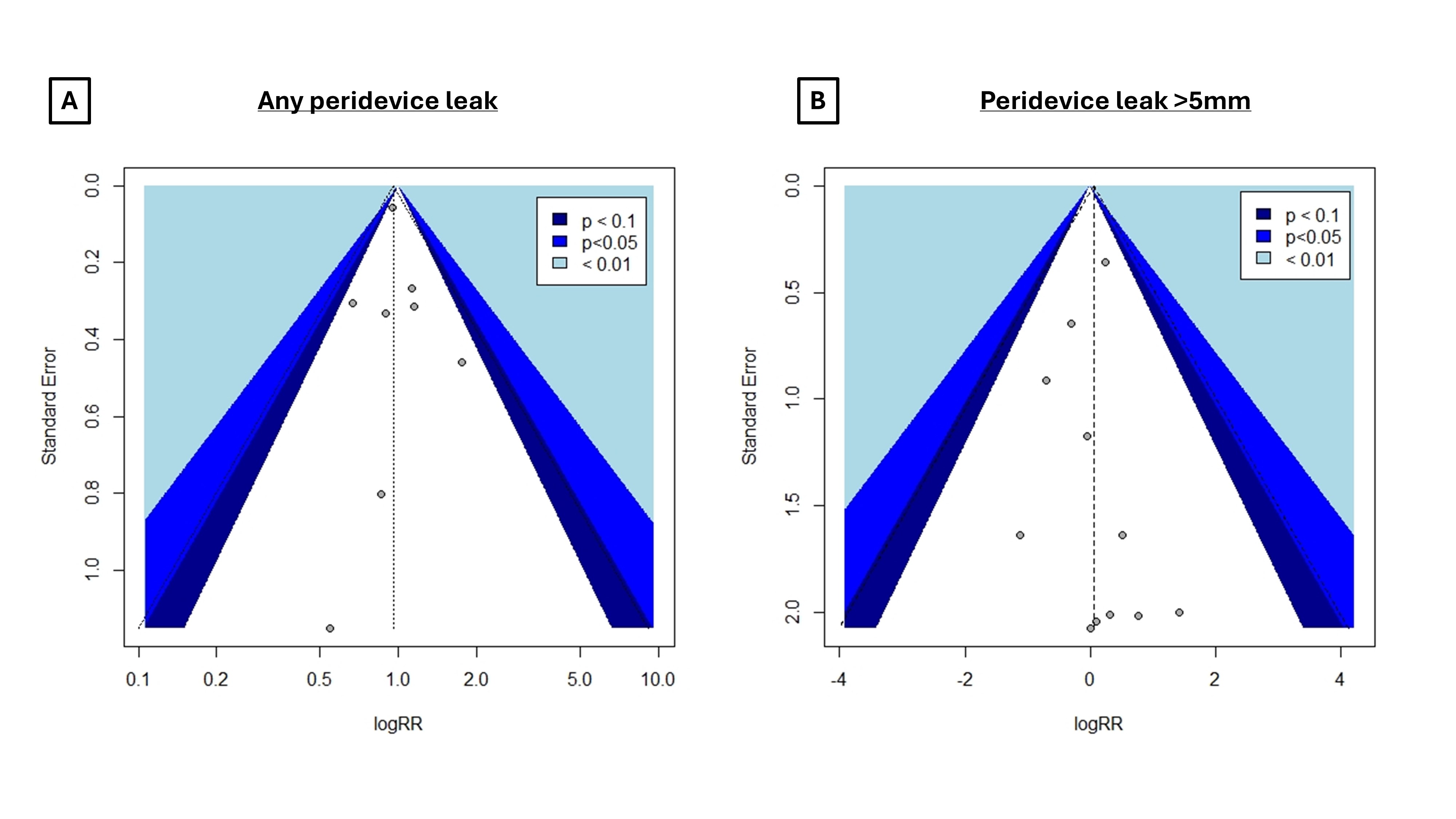

Supplement: Supplementary file 7 — Supplementary Material 7 [file 10554_2025_3330_MOESM7_ESM.jpg]

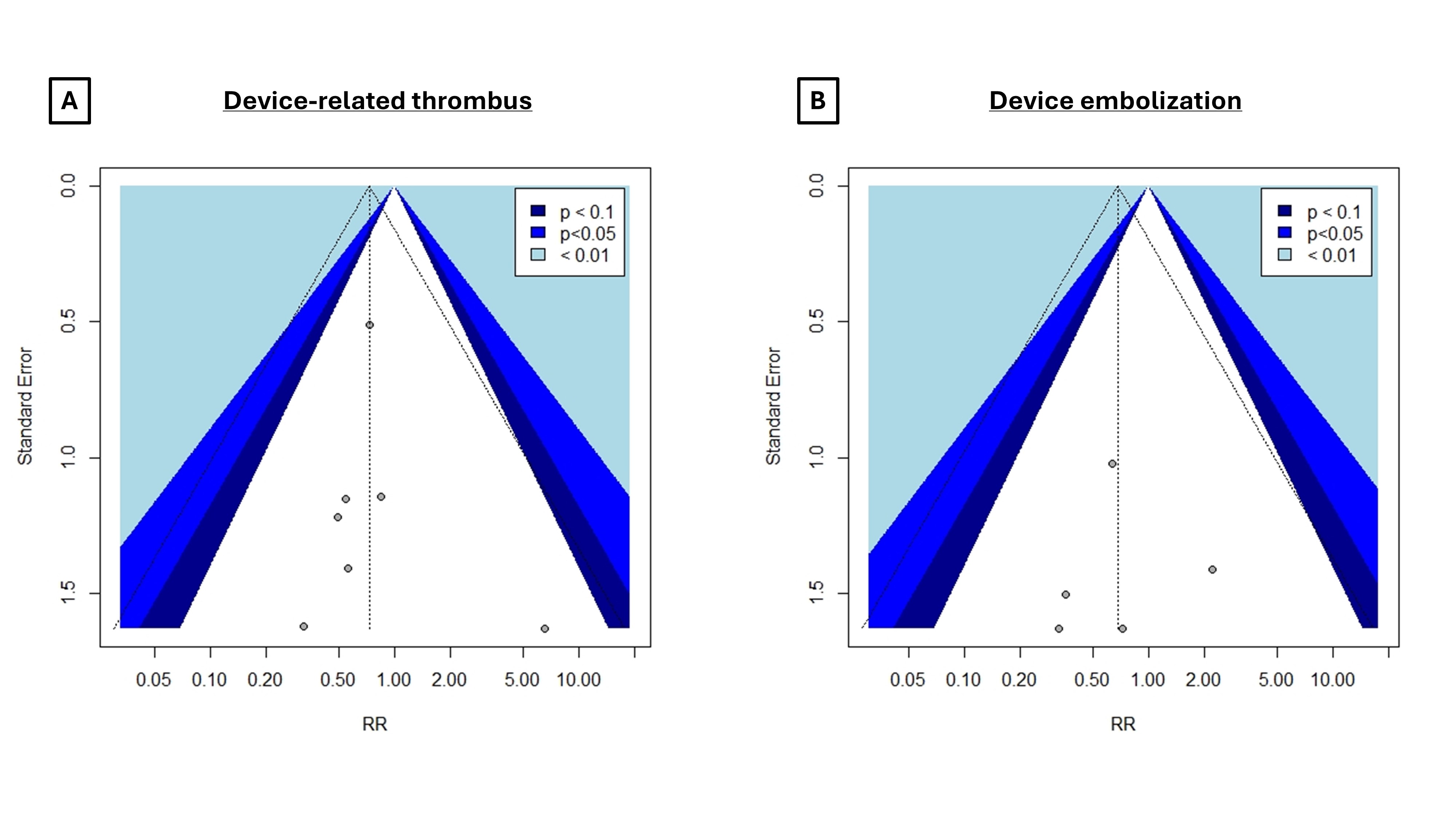

Supplement: Supplementary file 8 — Supplementary Material 8 [file 10554_2025_3330_MOESM8_ESM.jpg]
