# Supplementary material for: Intracardiac or transesophageal echocardiography for left atrial appendage occlusion: an updated systematic review and meta-analysis
Source: Int J Cardiovasc Imaging. 2025 Jan 22;41(3):489–505. doi: 10.1007/s10554-025-03330-z (PMC11880089; doi:10.1007/s10554-025-03330-z)
Supplement: Supplementary file 9 — Supplementary Material 9 [file 10554_2025_3330_MOESM9_ESM.docx]

| **Supplementary Table. Publication bias assessed by Egger’s regression test.** | | | |
| --- | --- | --- | --- |
| **Outcome** | **Intercept** | **95% CI** | **p** |
| Technical success | -0.214 | -0.68, 0.25 | 0.41 |
| Devices used | 1.102 | -2.76, 4.96 | 0.61 |
| Procedure time | -1.28 | -3.91, 1.36 | 0.36 |
| Fluoroscopy time | -3.76 | -7.65, 0.14 | 0.09 |
| Contrast medium volume | -0.25 | -2.65, 2.16 | 0.85 |
| Device-related adverse events | 0.21 | -1.11, 1.53 | 0.77 |
| Pericardial effusion | -0.584 | -1.19, 0.02 | 0.08 |
| Iatrogenic atrial septal defect | 0.435 | -2.35, 3.22 | 0.79 |
| Vascular complications | -0.207 | -0.89, 0.48 | 0.57 |
| All bleeding | 0.221 | -0.36, 0.8 | 0.48 |
| Major bleeding | -0.553 | -1.17, 0.11 | 0.15 |
| Any peridevice leak | 0.091 | -0.66, 0.84 | 0.82 |
| Peridevice leak >5mm | -0.047 | -0.57, 0.47 | 0.86 |
| Device-related thrombus | 0.246 | -0.5, 0.99 | 0.54 |
| Device embolization | -0.079 | -0.98, 0.82 | 0.87 |
